# Compensation patterns and altered functional connectivity in alcohol use disorder with and without Korsakoff's syndrome

**DOI:** 10.1093/braincomms/fcae294

**Published:** 2024-09-20

**Authors:** Alexandrine Morand, Alice Laniepce, Nicolas Cabé, Céline Boudehent, Shailendra Segobin, Anne-Lise Pitel

**Affiliations:** Normandie Université, UNICAEN, INSERM, U1237, PhIND ‘Physiopathology and Imaging of Neurological Disorders’, Team NeuroPresage, Institut Blood and Brain @ Caen-Normandie, Cyceron, 14000 Caen, France; Laboratoire DysCo, Université Paris 8 Vincennes-Saint-Denis, 93526 Saint-Denis, France; Normandie Université, UNICAEN, INSERM, U1237, PhIND ‘Physiopathology and Imaging of Neurological Disorders’, Team NeuroPresage, Institut Blood and Brain @ Caen-Normandie, Cyceron, 14000 Caen, France; Normandie Université, UNIROUEN, CRFDP (EA 7475), 76000 Rouen, France; Normandie Université, UNICAEN, INSERM, U1237, PhIND ‘Physiopathology and Imaging of Neurological Disorders’, Team NeuroPresage, Institut Blood and Brain @ Caen-Normandie, Cyceron, 14000 Caen, France; Service d’addictologie, Centre Hospitalier Universitaire de Caen, 14000 Caen, France; Normandie Université, UNICAEN, INSERM, U1237, PhIND ‘Physiopathology and Imaging of Neurological Disorders’, Team NeuroPresage, Institut Blood and Brain @ Caen-Normandie, Cyceron, 14000 Caen, France; Service d’addictologie, Centre Hospitalier Universitaire de Caen, 14000 Caen, France; Normandie Université, UNICAEN, PSL Université Paris, EPHE, INSERM, U1077, CHU de Caen, Neuropsychologie et Imagerie de la Mémoire Humaine, GIP Cyceron, 14000 Caen, France; Normandie Université, UNICAEN, INSERM, U1237, PhIND ‘Physiopathology and Imaging of Neurological Disorders’, Team NeuroPresage, Institut Blood and Brain @ Caen-Normandie, Cyceron, 14000 Caen, France; Institut Universitaire de France (IUF), 75231 Paris, France

**Keywords:** alcohol use disorder, Korsakoff’s syndrome, resting-state functional imaging, fronto-cerebellar circuit, Papez circuit

## Abstract

Alcohol use disorder is a chronic disease characterized by an inappropriate pattern of drinking, resulting in negative consequences for the individual's physical, mental and social health. Korsakoff's syndrome is a complication of alcohol use disorder and is characterized by severe memory and executive deficits. The fronto-cerebellar and Papez circuits are structurally affected in patients with alcohol use disorder with and without Korsakoff’s syndrome. The first objective of the present study was to measure the effect of chronic and excessive alcohol consumption on resting-state functional connectivity of these two functional brain networks. The second objective was to identify, for the first time, resting-state functional connectivity abnormalities specific to amnesic patients with Korsakoff’s syndrome. In the present study, a neuropsychological assessment and a resting-state functional magnetic resonance imaging examination were conducted in 31 healthy controls (43.6 ± 6.1 years) and 46 patients (46.6 ± 9.1 years) with alcohol use disorder including 14 patients with Korsakoff’s syndrome (55.5 ± 5.3 years) to examine the effect of chronic and heavy alcohol consumption on functional connectivity of the fronto-cerebellar and the Papez circuits at rest and the specificity of functional connectivity changes in Korsakoff’s syndrome compared to alcohol use disorder without Korsakoff’s syndrome. The resting-state functional connectivity analyses focused on the nodes of the fronto-cerebellar and Papez circuits and combined region of interest and graph theory approaches, and whether these alterations are associated with the neuropsychological profile. In patients pooled together compared to controls, lower global efficiency was observed in the fronto-cerebellar circuit. In addition, certain regions of the fronto-cerebellar and Papez circuits were functionally hyperconnected at rest, which positively correlated with executive functions. Patients with Korsakoff’s syndrome showed lower resting-state functional connectivity, lower local and global efficiency within the Papez circuit compared to those without Korsakoff’s syndrome. Resting-state functional connectivity positively correlated with several cognitive scores in patients with Korsakoff’s syndrome. The fronto-cerebellar and Papez circuits, two normally well-segregated networks, are functionally altered by alcohol use disorder. The Papez circuit attempts to compensate for deficits in the fronto-cerebellar circuit, albeit insufficiently as evidenced by patients’ overall lower cognitive performance. Korsakoff’s syndrome is characterized by altered functional connectivity in the Papez circuit known to be centrally involved in memory.

## Teaser

Morand *et al*. examined resting-state functional connectivity within the Papez circuit and the fronto-cerebellar circuit in patients with alcohol use disorder and Korsakoff’s syndrome. They revealed between-network reorganisation in patients, as an attempt to compensate for connectivity abnormalities. Korsakoff’s syndrome is characterized by altered functional connectivity in the Papez circuit.

## Graphical Abstract

**Graphical Abstract:**
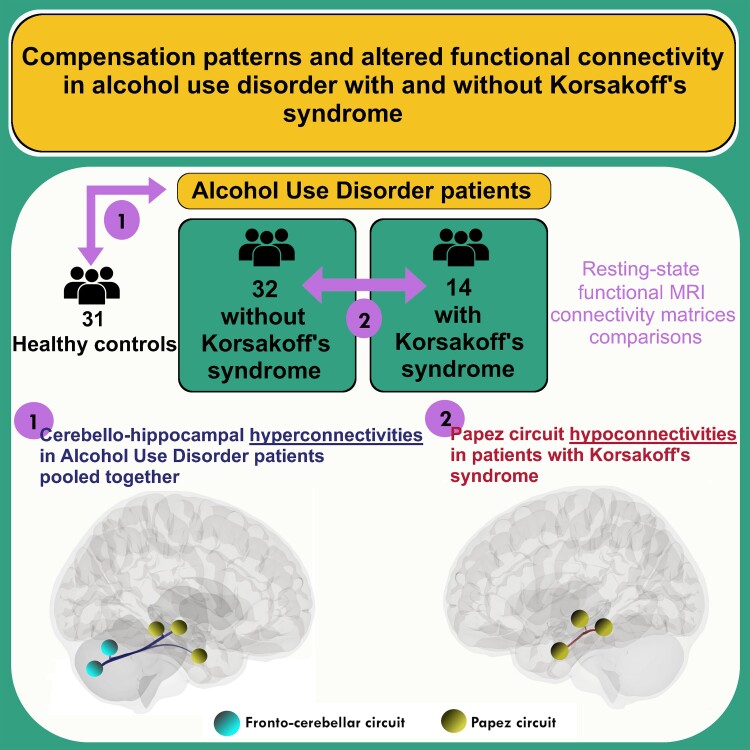

## Introduction

Alcohol use disorder (AUD) is a highly prevalent mental condition characterized by maladaptive alcohol consumption leading to significant impairment or distress.^1^ In some cases, heavy alcohol intake combined with thiamine deficiency can lead to Wernicke's encephalopathy (WE), progressing to Korsakoff's syndrome (KS) if untreated. KS is marked by severe and persistent anterograde amnesia, often accompanied by executive dysfunction, resulting in two clinical forms of AUD: with and without KS. Sometimes, AUD with and without KS is marked by ataxia associated with cerebellar damage.^2^

In both clinical forms of AUD, the fronto-cerebellar circuit (FCC) and the Papez circuit (PC) are affected.^3^ The FCC consists of two parallel loops: one crucial for motor abilities and another highly involved in working memory and executive functions.^4^ The PC primarily plays a role in episodic memory.^5^ Both brain circuits share the thalamus as a common region.

In AUD without KS, reduced grey matter volumes are observed in multiple brain regions of FCC and PC.^6,7^ Comparisons between AUD and KS patients indicate more severe shrinkage of the hippocampus^8^ and thalamus^9^ in KS patients. Neuropsychological comparisons reveal more pronounced episodic memory deficits in KS patients,^10^ while results for executive functions and working memory vary among studies, with some showing greater deficits in KS patients and others reporting similar alterations.^11,12^

Numerous studies emphasize the connection between resting-state neural activity organisation and cognitive performance.^13,14^ Limited information exists in AUD regarding functional connectivity within these networks and its correlation with neuropsychological performance. Chronic and excessive alcohol consumption is associated with inconsistencies in the resting-state functional connectivity (rs-FC) results. Indeed, while some studies report lower rs-FC in various networks,^15-17^ others report higher activity^18,19^ in patients with AUD compared to healthy controls (HC). The altered global brain synchronisation can be interpreted by different mechanisms as compensatory, recovery processes, network dedifferentiation, or pre-existing risk factors for AUD development.^20,21^ Compensatory mechanisms are correlated with cognitive performance, especially with executive functions, working memory and decision-making.^16,17^ The few studies that explored rs-FC in WE^22^ found lower connectivity in mammillothalamic and cerebellar regions in patients AUD recovering from WE compared to HC, which correlated with episodic memory or spatial memory.^23,24^ Nonetheless, brain functional connectivity at rest has never been explored at the chronic stage of KS.

The first aim of this study was thus to examine rs-FC in patients with AUD. Due to the heterogeneity of cognitive deficits and brain damage associated with AUD, we initially combined patients with and without KS and compared them to HC. The second objective was to directly compare patients with and without KS, to highlight the specificities of rs-FC abnormalities in KS. This marks the first exploration of rs-FC in a relatively large group of KS patients. Our analyses focused on regions belonging to the PC and FCC using regions of interest (ROI-to-ROI) and graph theory methods. Additionally, we investigated the relationships between rs-FC measures and cognitive performance.

## Materials and methods

### Population

Forty-six patients (32 men, 14 women) with severe AUD (DSM-5 criteria, American Psychiatric Association, 2013) and 31 HC (25 men, 6 women) were included in the study. The demographical and clinical data of participants are available in Table 1. This study was approved by the Nord-Ouest III French Ethics Committee and carried out in line with the Declaration of Helsinki (1964). All participants gave their informed written consent to the study.

**Table 1:** Demographical, clinical and cognitive description of the healthy participants and patients with AUD and Korsakoff’s syndrome

	HC (*n* = 31)	AUD (*n* = 32)	KS (*n* = 14)	*F*	Group differences
Demographic data					
Gender (M/W)	25/6	28/4	4/10		(HC = AUD)≠KS
Age (years)^a^	43.6 ± 6.1 [31–55]	46.6 ± 9.1 [26–63]	55.5 ± 5.3 [44–63]	7.37	(HC = AUD)<KS
Education (years)	11.6 ± 1.6 [9–15]	12.0 ± 9.0 [9–17]	10.0 ± 2.3 [6–15]	4.64	(HC = AUD)>KS
AUDIT	2.7 ± 1.4 [0–6]	28.2 ± 6.2 [9–38]	N/A	543.84	HC < AUD
BDI	3.5 ± 3.8 [0–14]	11.7 ± 7.7 [1–34]	10.0 ± 8.2 [0–29]	12.37	HC < (AUD = KS)
STAI A	27.0 ± 6.4 [20–47]	29.9 ± 9.3 [20–67]	34.7 ± 14.6 [20–66]	4.30	HC = AUD = KS
STAI B	33.3 ± 7.3 [20–50]	44.9 ± 11.8 [28–68]	40.5 ± 10.9 [24–57]	10.84	HC < (AUD = KS)
Fagerström	1.0 ± 2.0 [0–7]	3.97 ± 3.3 [0–6]	2.21 ± 2.0 [0–14]	11.35	HC < (AUD = KS)
Clinical data					
Abstinence before inclusion (days)	N/A	10.9 ± 4.8 [4–24]	N/A		/
AUD (years)	N/A	21.7 ± 10.6 [3–42]	N/A		/
Number of previous detoxifications	N/A	1.9 ± 2.2 [0–11]	N/A		/
Neuropsychological data					
Global cognitive evaluation					
MMSE (/30)	28.5 ± 1.1 [27–30]	27.5 ± 1.8 [25–30]	23.4 ± 2.9 [18–27]	19.44	(HC = AUD)>KS
Mattis dementia rating s cale (/144)	141.9 ± 2.0 [136–144]	137.2 ± 5.9 [119–143]	118.4 ± 11.4 [95–133]	52.93	(HC = AUD)>KS

HC, healthy controls; AUD, patients with alcohol use disorder without Korsakoff’s syndrome; KS, Patients with Korsakoff‘s syndrome; M, men; W, women; AUDIT, alcohol use disorders test; BDI, Beck depression inventory; MMSE, mini-mental state examination; STAI, state-trait anxiety inventory for adults (A = state-anxiety and B = trait-anxiety); N/A, not available.

Mean ± standard deviation and range [minimum–maximum] are reported.

ANCOVA with age and gender as a covariate. *Post hoc* HSD Tukey was used at *P* < 0.05.

^a^ANCOVA with gender as a covariate.

Of the 46 patients with AUD, 14 (4 men and 10 women) were diagnosed with KS. Patients with KS were recruited at Caen University Hospital (*n* = 6) and in a nursing home (Maison Vauban, Roubaix, France; *n* = 8). All patients with KS had a long history of chronic and heavy drinking. Each patient was rigorously selected and examined by a multi-disciplinary team made up of specialists in cognitive neuropsychology and behavioural neurology. A detailed neuropsychological examination enabled the diagnosis of KS (Table 2).

**Table 2:** Neuropsychological performance (*Z*-scores) of the healthy participants and patients with AUD and Korsakoff’s syndrome

	HC (*n* = 31)	AUD (*n* = 32)	KS (*n* = 14)	*F*	Group differences
Memory					
Verbal episodic memory					
FCRST sum of the three free recalls	0 ± 1.0 [−2.47/1.45]	−0.98 ± 1.0 [−4.86/0.42]	−3.44 ± 1.60 [−5.71/−0.26]	10.77	HC > AUD > KS
FCRST delayed free recall	0 ± 1.0 [−2.16/1.45]	−1.07 ± 1.3 [−5.26/0.42]	−5.15 ± 1.68 [−6.80/−1.13]	9.22	HC > AUD > KS
CVLT sum of the five free recalls	0 ± 1.0 [−2.29/1.37]	N/A	−5.64 ± 1.13 [−7.47/−3.81]	98.37	HC > KS
CVLT delayed free recall	0 ± 1.0 [−2.81/0.99]	N/A	−7.06 ± 0.70 [−7.69/−5.52]	275.34	HC > KS
Visual episodic memory					
ROCFT immediate recall score (/36)	0 ± 1.0 [−2.18/1.82]	−0.41 ± 1.0 [−2.84/1.15]	−2.22 ± 0.60 [−2.84/−0.71]	13.61	(HC *=* AUD)>KS
ROCFT delayed recall score (/36)	0 ± 1.0 [−2.59/1.91]	−0.37 ± 1.15 [−2.94/2.19]	−2.36 ± 0.61 [−2.94/−1.02]	15.00	(HC *=* AUD)>KS
Memory composite *Z*-score	0 ± 1.0 [−1.93/1.23]	−0.70 ± 0.84 [−3.18/0.42]	−4.37 ± 0.56 [−5.29/−3.55]	106.36	HC > AUD > KS
Visuospatial abilities					
ROCFT copy score (/36)	0 ± 1.0 [−2.84/0.60]	−0.91 ± 2.12 [−6.28/0.60]	−2.42 ± 4.14 [−14.53/0.60]	6.36	(HC = AUD)>KS
Visuospatial abilities composite *Z*-score	0 ± 1.0 [−2.84/0.60]	−0.91 ± 2.12 [−6.28/0.60]	−2.42 ± 4.14 [−14.53/0.60]	6.36	HC > AUD > KS
Executive functions					
Inhibition					
STROOP interference-naming condition (in seconds)	0 ± 1.0 [−4.05/1.24]	−0.52 ± 1.14 [−4.48/1.19]	−1.91 ± 3.16 [−11.7/0.59]	5.27	(HC = AUD)>KS
Shifting					
TMT part B-A (in seconds)	0 ± 1.0 [−2.91/1.66]	−1.52 ± 2.77 [−9.64/1.55]	−5.13 ± 4.86 [−13.75/0.75]	12.73	HC > AUD > KS
MCST number of perseverative errors	0 ± 1.0 [−0.71/3.45]	0.52 ± 1.73 [−0.71/5.83]	1.72 ± 2.00 [−0.71/5.83]	3.78	HC = AUD > KS
Executive functions composite *Z*-score	0 ± 1.0 [−1.83/0.93]	−0.50 ± 1.06 [−3.79/1.13]	−2.11 ± 2.27 [−6.54/0.62]	12.18	HC > AUD > KS
Processing speed					
TMT part A (in seconds)	0 ± 1.0 [−2.58/1.45]	−1.59 ± 1.42 [−4.79/0.64]	−4.14 ± 3.68 [−13.25/−0.67]	14.84	HC > AUD > KS
STROOP naming condition (in seconds)	0 ± 1.0 [−2.07/1.91]	−1.43 ± 2.22 [−11.01/0.84]	−3.13 ± 3.84 [−15.08/−0.23]	13.14	HC > AUD > KS
Processing speed composite *Z*-score	0 ± 1.0 [−1.67/1.48]	−1.48 ± 1.66 [−7.90/0.49]	−3.63 ± 3.29 [−12.96/−0.64]	17.87	HC > AUD > KS

HC, healthy controls; AUD, patients with alcohol use disorder without Korsakoff’s syndrome; KS, patients with Korsakoff‘s syndrome; FCRST, free and cued recall reminding test; CVLT, California verbal learning test; ROCFT, Rey–Osterrieth complex figure test; TMT, trail making test; MCST, modified cart sorting test.

Mean *Z*-score ± standard deviation and range [minimum/maximum] are reported.

ANCOVA with age and gender as covariate. *Post hoc* HSD Tukey was used at *P* < 0.05.

The 32 other patients with AUD (28 men, 4 women), without KS, were recruited at Caen University Hospital. Patients were early in abstinence (16.7 ± 21.4 days of sobriety prior to inclusion), and none of them had physical symptoms of alcohol withdrawal as assessed by the Cushman’s scale at inclusion. They were interviewed with a modified version of the semi-structured lifetime drinking history and the alcohol use disorders identification test (AUDIT). Measures included the duration of alcohol use, AUD, number of previous detoxifications and daily alcohol consumption prior to treatment (in units, a unit corresponding to a beverage containing 10 g of pure ethanol) (see Table 1).

The HC group (*n* = 31; 25 men, 6 women) was recruited locally and matched the demographics of the entire AUD group. Inclusion criteria were: a score above 26 at the mini-mental state examination (MMSE) or a score above 129 at the Mattis Dementia Rating score, and a score below 6 for females and 7 for males at the AUDIT (Table 1).

To be included, all participants had to be between 18 and 70 years old, and to have French as their native language. No participant had a co-morbid psychiatric disorder, was taking psychotropic medication, had a history of serious chronic pathology, neurological problems that might have affected cognitive function, or had taken other substances (except tobacco and alcohol for the patients).

AUD and HC were matched for gender, age and education. Patients with KS differed from both HC and AUD on gender, age and education. Age, education, MMSE/Mattis scores,^25,26^ depression (BDI),^27^ and anxiety scores (state-trait anxiety inventory, STAI)^28^ as well as nicotine dependence severity level (Fagerström score)^29^ are reported in Table 1.

### Neuropsychological assessment

#### Memory

‘Verbal episodic memory’ was assessed with the French version of the free and cued selective reminding test^30^ (FCSRT; adapted from Grober and Buschke^31^) for all participants, except KS patients who performed the California verbal learning test^32^ (CVLT). We used the sum of the three free recalls (score/48) and the delayed free recall (score/16) of the FCSRT and the sum of the five free recalls (score/80) and the delayed free recall (score/16) of the CVLT. ‘Visual episodic memory’ was assessed by the immediate and delayed recalls of the Rey–Osterrieth complex figure^33^ (ROCF; score/36).

Visuospatial abilities were assessed by the copy of the ROCF (score/36).

#### Executive functions

‘Inhibition’ was assessed by the Stroop task^34^ (time for interference condition minus time for naming condition). ‘Shifting’ was assessed with the trail making test^35^ (TMT; time for Part B minus time for Part A). It was also assessed with the number of perseverations at the modified cart sorting test^36^ (MCST).

‘Processing speed’ was assessed by the time to complete the Part A of the TMT and the naming condition of the Stroop test.

### Neuroimaging data acquisition

#### Anatomical MRI

A high-resolution 1 mm^3^ T1-weighted anatomical image was acquired for each participant on a 3T MR Philips imager (Achieva 3.0 T TX) at the Cyceron neuroimaging centre (Caen, France) using a three-dimensional fast-field echo sequence (repetition time = 20 ms, echo time = 4.6 ms, flip angle = 10°, 180 slices, slice thickness = 1 mm, field of view = 256 × 256 mm² and matrix = 256 × 256).

#### Resting-state functional MRI data

Rs-fMRI data were acquired using an interleaved bottom to top 2D T2* SENSitivity Encoding EPI sequence designed to reduce geometric distortions (2D-T2*-FFE-EPI axial using 80 × 80 × 52 axial slices, 2.8 mm thickness, TR = 2.90 s, TE = 30 ms, flip angle = 80°, field of view = 224 × 224, no gap, in-plane voxel size = 2.8 × 2.8 mm^2^ and 240 volumes and acquisition time = 11.6 min).

### Neuroimaging data processing

#### Resting-state functional MRI data processing

##### Spatial preprocessing

Rs-fMRI data (also called EPI volumes) were processed using the functional connectivity toolbox^37^ (CONN toolbox; https://www.nitrc.org/projects/conn). The CONN toolbox uses the statistical parametric mapping software (SPM12; Welcome Department of Cognitive Neurology, Institute of Neurology, London, UK) implemented in MATLAB (R2021) (MathWorks Inc., Natick, MA) (see Supplementary Fig. 1 for spatial pre-processing procedure). Steps were: (i) artifacts removing using the artifact detection tools (ART tools; https://www.nitrc.org/projects/artifact_detect) and slice timing correction, (ii) normalisation to reduce geometrical distortions effects,^38^ (iii) coregistration including the alignment between the mean EPI and the non-EPI T2* volumes, then between the non-EPI T2* and T1 volumes, (iv) normalisation of the mean EPI volume to match the non-EPI T2* volume, (v) segmentation of T1 volume,^39^ (vi) normalisation of the coregistered T1, non-EPI T2* and EPI volumes to matching analogous anatomical regions in a common space (the Montreal Neurological Institute template) and (vii) smoothing of EPI volumes using a 4 mm full-width at half-maximum Gaussian kernel. Following steps included temporal reduction preprocessing (denoising step), first-level (individual) analysis and second-level (group) analysis.

##### Temporal preprocessing (denoising step)

The denoising step was applied on the previously preprocessed rs-fMRI data in order to minimize the influence of physiological sources, and reduce inter-subject variability. Noise reduction procedure in CONN toolbox used the anatomical component-based noise correction (aCompCor).^40^ EPI volumes were filtered with the covariates from the T1 volume segmentation, including the first five principal components of WM and CSF. Motion parameters (six motion parameters and their first and second derivatives, totalling 18 parameters) from SPM and ART-generated variables (scrubbing and framewise displacement) were also included as covariates. After regressing, temporal frequencies below 0.008 Hz or above 0.09 Hz were removed from the resulting blood oxygenation level dependent time series to reduce noise and increase the sensitivity of measures.^41^

### Statistical analyses

#### Resting-state functional connectivity analysis

##### First-level analyses

Brain regions were identified using the Harvard–Oxford probabilistic anatomical brain atlas.^42^ For each participant, bivariate Pearson’s correlation coefficients were calculated in the CONN toolbox for every possible pairs of regional time series. Of the 132 atlas ROI, 18 ROIs were considered for analyses with a total of 153 possible pairs of regions. According to the objectives of the present study, we chose (bilaterally):

- Middle frontal gyrus (BA46), superior frontal gyrus (BA9), cerebellum Crus I (Cereb1), cerebellum Crus II (Cereb2) for the executive loop of the FCC;
- Hippocampus (Hipp), anterior parahippocampal cortex (aPaHC) corresponding to entorhinal cortex, posterior parahippocampal cortex (pPaHC), anterior cingulate gyrus and posterior cingulate gyrus for the PC;
- Thalamus (Thal) involved in both networks (see Supplementary Fig. 2).

Correlation coefficients were converted to normally distributed scores using Fisher *Z*-transformation. The resultant 18 × 18 matrices have been used for second-level group comparison statistical analyses described below.

##### Second-level analyses

To meet our first objective, a ROI-to-ROI approach was used to compare rs-FC between the patients’ group (patients with AUD and KS pooled together) and the HC group. We used two-sample *t*-tests with the hypothesis of a lower (i.e. hypoconnectivity) or higher (i.e. hyperconnectivity) in rs-FC in the patient group compared to HC controlling for age and gender. ROI-to-ROI comparisons were corrected for multiplicity and are reported for *P* < 0.05, family wise error (FWE).

To meet our second objective, differences in rs-FC between patients with AUD and KS were explored, using the same statistical approach described above (two-sample *t*-tests on 18 × 18 ROI-ROI matrix). Results are reported for a statistical threshold that is less stringent (*P* < 0.001 uncorrected for multiple comparisons).

##### Graph theory analysis

All ROI-to-ROI measures were based on graphs with nodes (ROI) and edges (connections). For each subject, a graph adjacency matrix was calculated by thresholding the associated ROI-to-ROI correlation matrix. Two main measures were computed to address topological properties information for each network of ROIs: the local efficiency (Eloc) and global efficiency (Eglob). These two measures represent networks segregation and networks integration, respectively.^43,44^ The local efficiency, $\mathrm{Eloc}=\;\frac{1}{n}\sum_{i\in G}\;\mathrm{Eglob}(Gi)$, illustrates the communication propagation efficiency within interconnected regions where *Gi* is the local subgraph obtained by the neighbours of node *i*, Eglob(Gi) is the global efficiency of *Gi*; i.e. the higher the value, the better is the local efficiency and the network is less segregated. The global efficiency, $lob=\;\frac{1}{n(n-1)}\;\sum_{\begin{array}{c} ij\in G\\ i\neq j \end{array}}\;\frac{1}{d\;ij}$, represents the average communication efficiency between a node and all other nodes in the same network where *n* is the total number of nodes in the network *G*, and *dij* is the minimum average number of links (length of the shortest path) that connect the node *i* and the node *j*; i.e. the higher the value, the faster is the flow of information, and the more the network is integrated. To keep the same statistical analysis strategy, we first analysed the differences on Eloc and Eglob for the FCC and the PC between the patient (AUD and KS pooled together) and HC groups using two-sample *t*-tests and then between AUD and KS groups.

Given the distribution and the difference in age and gender between groups, these two parameters were entered as confounding variables in analyses.

### Relationships with neuropsychological performance

For each participant, neuropsychological results were transformed into *Z*-scores, using the mean and standard deviation of the HC group (reported in Table 2). For each participant, four cognitive composite *Z*-scores were then computed:

- A composite *Z*-score for episodic memory was calculated with the *Z*-scores of the sum of the free recall and delayed free recall at the FCRST (or CVLT for KS) and the *Z*-scores of immediate and delayed recalls at the ROCFT.
- A *Z*-score for visuospatial abilities was calculated with the copy score at the ROCFT.
- A composite *Z*-score for executive functions was computed with relevant *Z*-scores of the TMT, Stroop task and perseverative responses at the MCST.
- A composite *Z*-score for processing speed was computed with *Z*-scores of the TMT and Stroop task.

We carried out correlational analyses between these composite *Z*-scores and rs-FC measures (ROI-to-ROI and graph theory) using Pearson’s correlations in the patient group (AUD and KS pooled together). We only included the measures that significantly differed between patients and HC in the second level analysis (ROI-to-ROI group results). Then, we conducted correlations separately in AUD and KS groups including only the measures that significant differed between AUD and KS in the ROI-to-ROI specificity rs-FC analysis.

## Results

### Comparison of rs-FC between HC and patients (AUD and KS pooled together)

#### ROI-to-ROI

**Figure 1:**
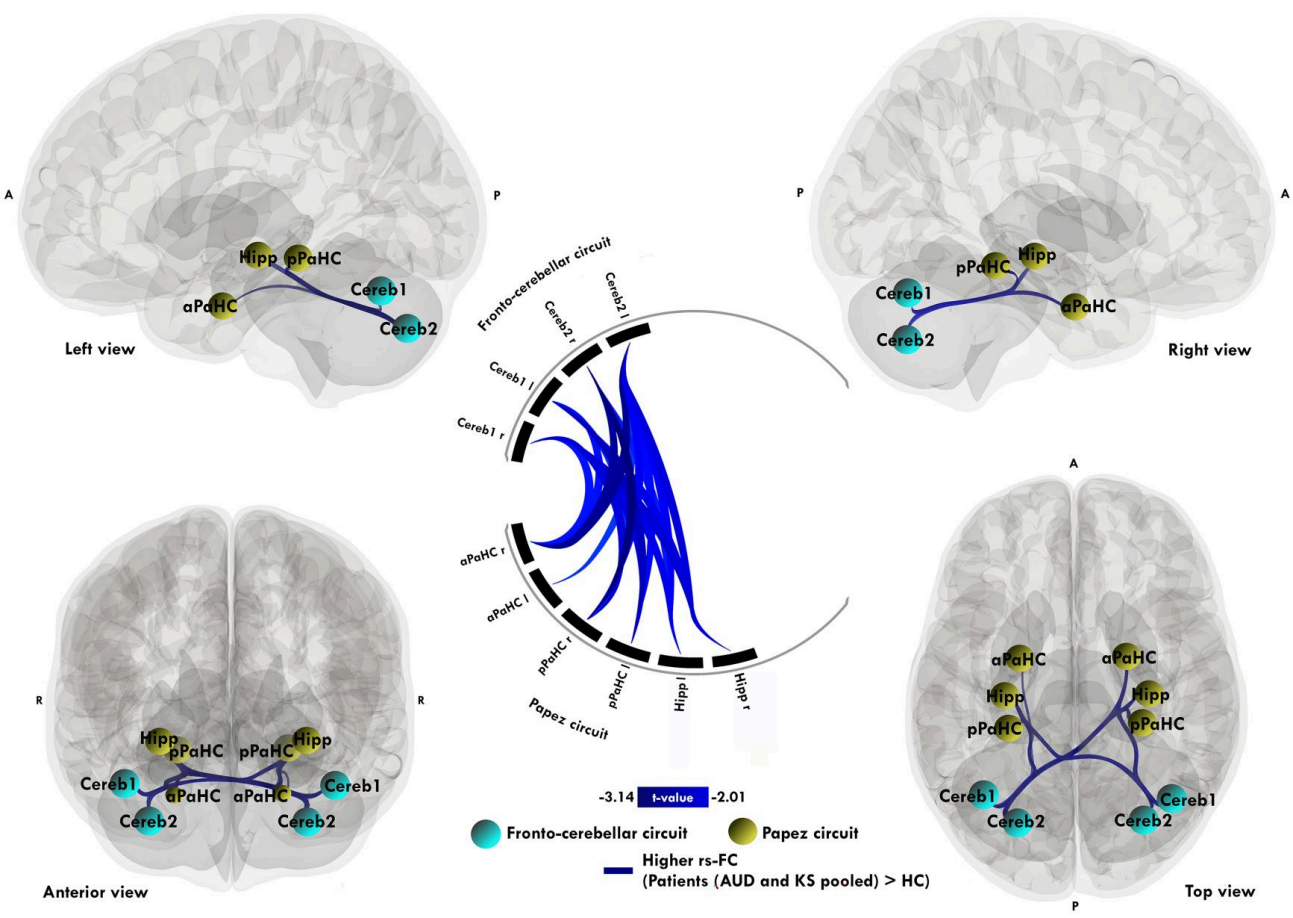
**Significant pairwise resting-state functional connectivity differences between patients (AUD and KS) and HC.** Significant ROI-to-ROI connectivity matrices comparisons with a two-sample *t*-test between patients (AUD and KS pooled together, *n* = 46) and HCs (*n* = 31). Blue links represent hyperconnectivity in patients (significant gain of functional connectivity, patients (AUD and KS pooled) > HC). Results are reported at *P* < 0.05 FWE. Regions are Cereb1, and Cereb2 included in the FCC; hippocampus (Hipp), anterior parahippocampal cortex (aPaHC), posterior parahippocampus (pPaHC) included in the PC.

Compared to HC, patients (AUD and KS pooled together) did not show lower rs-FC (*P* < 0.05 FWE) in the FCC and PC. For the opposite contrast, patients showed higher rs-FC compared to HC between the hippocampus, anterior parahippocampal cortex/entorhinal cortex and posterior parahippocampal cortex from the PC on the one hand and Cereb1 and Cereb2 on the other hand (*P* < 0.05 FWE). Results are displayed in Fig. 1. For more details on the statistic results, see Supplementary Table 1. For information, we have performed group comparisons without combining the AUD and KS groups: AUD patients and the HC, and KS patients and the HC. Results are displayed in Supplementary Fig. 3.

#### Graph theory: local and global efficiency

**Figure 2:**
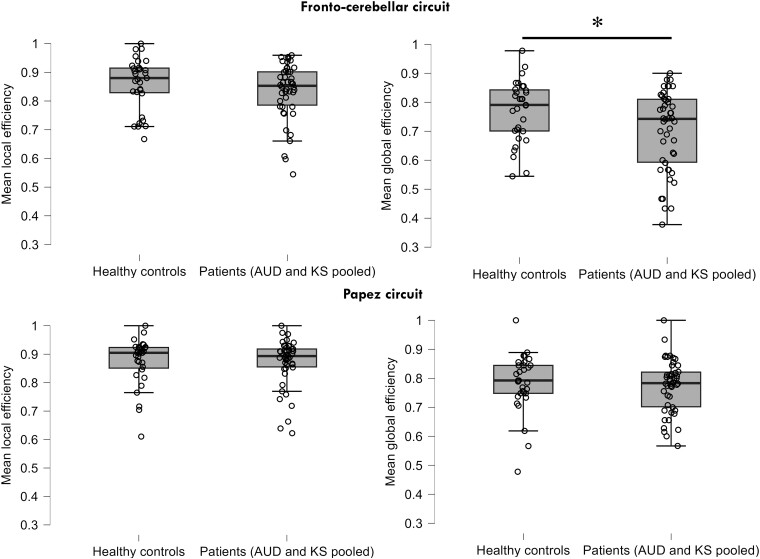
**Graph theory results for the fronto-cerebellar and PCs in patients (AUD and KS) and HC.** Significant differences on mean local and global efficiency for the fronto-cerebellar and PCs between patients (AUD and KS pooled together, *n* = 46) and HC (*n* = 31) groups using two-sample *t*-tests. *Significant *P* < 0.05.

Compared to HC, patients had lower global efficiency (*t* = 2.35, *P* = 0.02) measures in the FCC (Fig. 2). We did not observe any between group differences for the local efficiency in the FCC (*t* = 1.15; *P* = 0.26). No significant differences were observed for the PC (Eglob: *t* = 0.50; *P* = 0.62; Eloc: *t* = 0.18; *P* = 0.86).

### Comparison of rs-FC between AUD and KS patients

#### ROI-to-ROI

**Figure 3:**
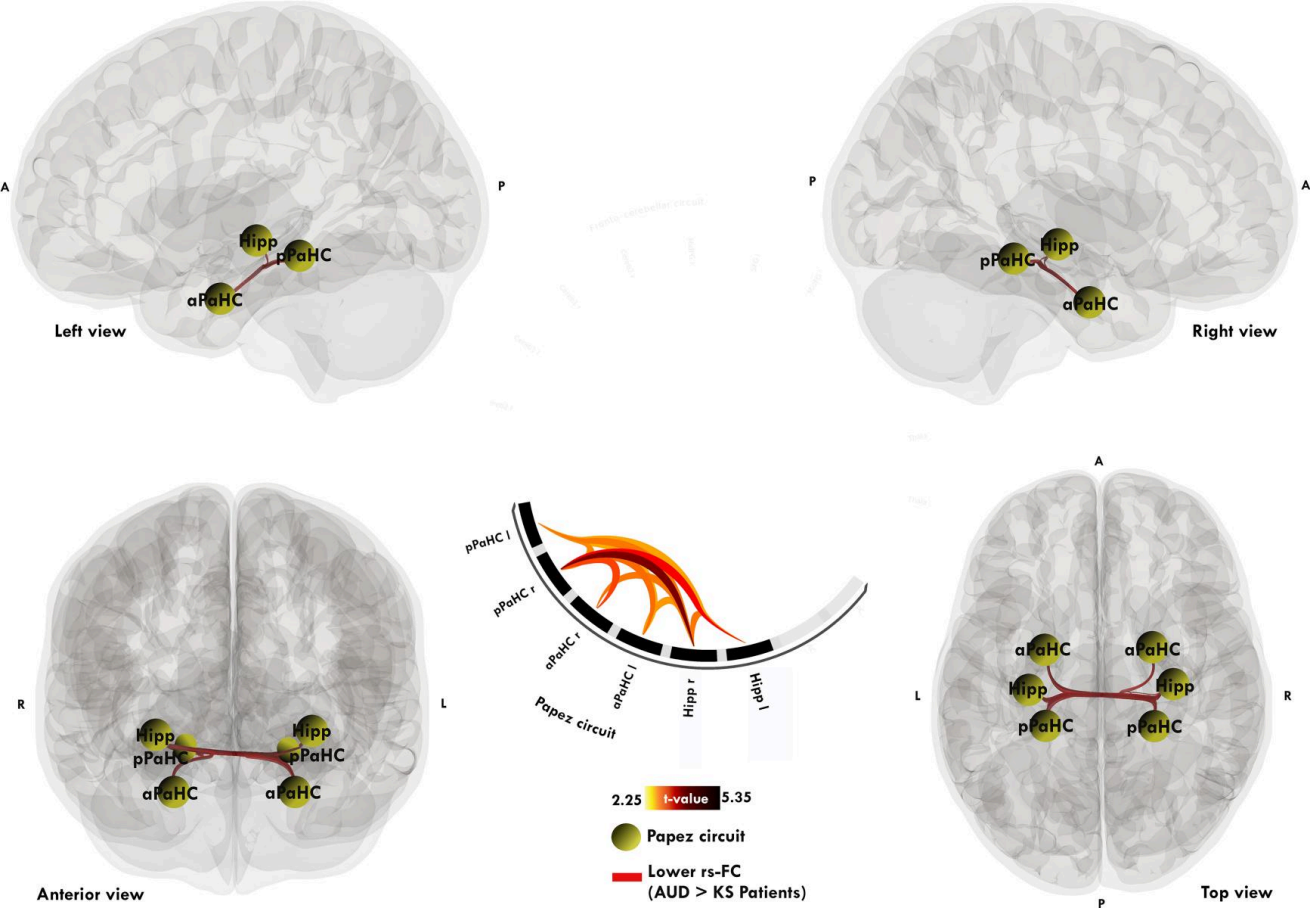
**Significant pairwise resting-state functional connectivity differences between patients with KS and AUD.** Significant ROI-to-ROI connectivity matrices comparisons with a two-sample *t*-test between patients with KS (*n* = 14) and AUD (*n* = 32). Red links represent hypoconnectivity in patients with KS (significant loss of functional connectivity, AUD patients > KS patients). Results are reported at *P* < 0.001 uncorrected. Regions are: hippocampus (Hipp), anterior parahippocampal cortex (aPaHC), and posterior parahippocampal cortex (pPaHC) called PC.

Compared to patients with AUD, KS patients showed lower rs-FC between the anterior parahippocampi, hippocampi and posterior parahippocampi (Fig. 3, *P* < 0.001 uncorrected). For the opposite contrast, no between-group difference was observed. For more details, see Supplementary Table 2.

#### Graph theory: local and global efficiency

**Figure 4:**
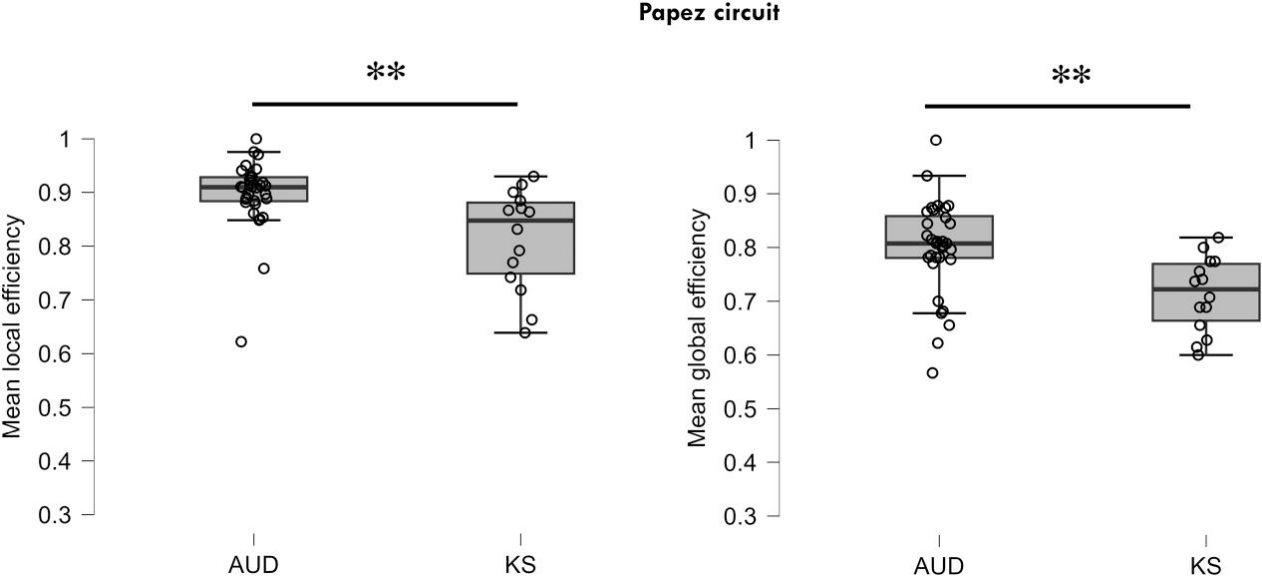
**Graph theory results for the PC in patients with KS and AUD.** Significant differences on mean local and global efficiency for the fronto-cerebellar and PCs between the patient KS (*n* = 14) and AUD (*n* = 32) groups using two-sample *t*-tests. **Significant *P* < 0.01.

Compared to AUD, KS patients had lower local (*t* = 3.43, *P* = 0.001) and global efficiency (*t* = 3.20, *P* = 0.003) measures in the PC (Fig. 4) but did not differ on the measures for the FCC (Eloc: *t* = −1.74, *P* = 0.087; Eglob: *t* = 1.76, *P* = 0.085).

### Correlations between resting-state functional connectivity measures (ROI-to-ROI and graph theory) and neuropsychological performance

Compared to HC, patients (AUD and KS pooled together) had lower performance on all cognitive domains. Patients with KS also had lower performances than patients with AUD on all measures. Composite *Z*-scores between-groups comparisons are reported in Table 2.

### Correlations between neuropsychological performance and ROI-to-ROI measures

#### AUD and KS pooled together

In the patient group (AUD and KS pooled together), ‘executive’ *Z*-score positively correlated with rs-FC measures between the right posterior parahippocampal cortex and the left Cereb2 (*r* = 0.40, *P* = 0.005). No other correlations were found between rs-FC measures and ‘episodic memory, processing speed or visuospatial’ composite scores.

#### AUD and KS separately

In AUD without KS, the ‘executive’ *Z*-score positively correlated with rs-FC between: right hippocampus and right posterior parahippocampal cortex (*r* = 0.39, *P* = 0.029), right hippocampus and left posterior parahippocampal cortex (*r* = 0.39, *P* = 0.026), left hippocampus and right hippocampus (*r* = 0.56, *P* = 0.001), left hippocampus and left posterior parahippocampal cortex (*r* = 0.36, *P* = 0.042), and left posterior parahippocampal cortex and left anterior parahippocampal cortex (*r* = 0.49, *P* = 0.004). The ‘episodic memory’ *Z*-score positively correlated with rs-FC between right hippocampus and left posterior parahippocampal cortex (*r* = 0.40, *P* = 0.025). The ‘processing speed’ *Z*-score positively correlated with rs-FC between: right posterior parahippocampal cortex and left anterior parahippocampal cortex (*r* = 0.47, *P* = 0.007), left posterior parahippocampal cortex and left anterior parahippocampal cortex (*r* = 0.62, *r* = 0.001), right hippocampus and left hippocampus (*r* = 0.53, *P* = 0.002), and right hippocampus and left anterior parahippocampal cortex (*r* = 0.37, *P* = 0.044). No correlations were found between rs-FC measures and the ‘visuospatial’ score.

In KS, no correlation was observed between rs-FC measures and ‘episodic memory’ *Z*-score. One negative correlation was found between the ‘executive’ *Z*-score and connectivity between the right anterior parahippocampal cortex and the right posterior parahippocampal cortex (*r* = −0.63, *P* = 0.016).

### Correlation with graph theory measures

#### AUD and KS pooled together

In the patient group, positive correlations were found between global efficiency (*r* = 0.40, *P* = 0.006) and local efficiency (*r* = 0.42, *P* = 0.003) in the PC and the ‘memory’ *Z*-score. We did not find any other correlations.

#### AUD and KS separately

In KS, a positive correlation was found between local efficiency in PC and the ‘executive’ *Z*-score (*r* = 0.62, *P* = 0.017).

In AUD, positive correlations were found between local efficiency in PC and the ‘executive’ (*r* = 0.49, *P* = 0.004) and ‘visuospatial abilities’ (*r* = 0.37, *P* = 0.038) *Z*-scores.

## Discussion

The first aim was to examine the effect of AUD on rs-FC within the FCC and PC in patients with and without KS compared to HC. In accordance with previous studies,^16,45^ we observed an effect of chronic and excessive alcohol consumption on rs-FC in patients with AUD pooled together compared with HC with hyperconnectivities between the cerebellum and hippocampal regions. In HC, rs-FC of cerebellar cruses correlated negatively with parahippocampi and hippocampi while the inverse correlations were observed between these same regions in patients with AUD pooled together.

These hyperconnectivities in rs-FC may reflect two brain mechanisms: dedifferentiation^46^ or compensation.^47,48^ Considering a pathological mechanism of dedifferentiation, the hyperconnectivities would indicate the inherent difficulty to recruit specific networks causing the recruitment of regions out of the network.^49^ These results would converge with another study in patients with AUD using a dynamic rs-FC approach, which found that the cerebellum tends to connect with other regions outside their ‘core’ functional systems, reflecting a pathological functional reorganisation.^50^ This hypothesis is supported by the process of neural dedifferentiation,^51^ which is defined as brain activity becoming increasingly distributed between neuronal populations that overlap and becomes less distinct from one another. To support this hypothesis, two conditions must be met: a reduced within-network connectivity and an increased connectivity between networks that are normally unrelated.^52^ This mechanism has been observed in several normal aging studies that found age-related reductions of within-network rs-FC and age-related increases of between-network rs-FC.^14,51,53^ In the present study, only an increased connectivity between-networks was found, no lower within-network connectivity, which validates one but not both conditions, hence not supporting the dedifferentiation hypothesis.

Alternately, the hyperconnectivities observed in the patient group could reflect a compensatory mechanism that would counteract pathology-related structural alterations.^6,7,9^ According to Cabeza *et al*.,^54^ two basic criteria must be fulfilled to attribute any greater activity to compensation. First, increased connectivity should be linked to a neural resource deficiency (e.g. atrophy, lower cerebral perfusion). Alterations in grey matter macrostructure and white matter microstructure are well described in patients with and without KS^55-57^ and have also been demonstrated in previously published studies using subsets of the cohort from this study.^58-60^ Second, the increase should correlate positively with cognitive performance, enabling us to differentiate compensation from non-specific and maladaptive recruitment (i.e. dedifferentiation), in which greater connectivity is not associated with better performance. Consistent with this last criterion, we found positive correlations between hyperconnectivity and executive performance. However, executive abilities remained impaired in the overall group of patients, suggesting an attempt at, but still not sufficient, compensation for all patients to achieve statistically comparable levels of cognitive performance compared to HC.

Graph theory analyses can provide an additional criterion to investigate resting-state compensation.^61^ Global efficiency is associated with long-range connections to ensure effective information transfer across distant regions. Local efficiency is mainly used in short-range connections among neighbouring regions to mediate alternative information processing. Our results indicate that the connectivity measures between FCC and PC are negatively related with global efficiency of FCC (Supplementary Table 3) in patients with AUD pooled together: lower is the global efficiency of the FCC, stronger is the rs-FC between the two networks. This finding indicates that AUD may be associated with alterations of long-range connections (altered FCC global efficiency) but not with short-range connections (preserved FCC local efficiency). Hyperconnectivities would favour the exchange of information in the globality of the networks but this compensation would not be sufficient to perform on par with HC at the group level.

The second goal was to directly compare patients with and without KS to highlight the specificity of rs-FC abnormalities in these brain networks in patients with KS. Rs-FC within the FCC was similar in AUD patients with and without KS, providing incremental evidence to previous studies indicating that the two groups do not differ regarding macrostructure,^57^ nor white matter microstructure^59^ in this brain network. Contrary to comparisons between all patients with AUD pooled together and HC, we did not find any hyperconnectivities between the FCC and PC in patients with KS compared to AUD patients without KS. We can hypothesize that at this stage of the disease, severe functional and structural brain abnormalities^8,57^ may hamper patients with KS to recruit additional brain regions, in agreement with the absence of correlation between rs-FC measures in the FCC and cognitive abilities.

The present study reveals, for the first time, that KS is characterized by lower rs-FC between hippocampi and parahippocampi as well as lower global and local efficiency of the PC compared to AUD without KS. PC is known to be structurally affected in patients with KS^9,23,57^ with severe damage to the thalamus, mammillary bodies, fornix and cingulum.^9,57,59,62^ There is less consensus about the hippocampal region, with some studies showing volume deficits^8,63^ and others showing no change.^64,65^ However, while correlations were observed between memory abilities and several rs-FC measures in the PC in all patients with AUD pooled together, as well as in patients with AUD without KS, we did not find such relationships in the KS group. This absence of correlation in patients with KS may be explained by the choice of the memory score or the overall very low memory performance in this group, with small variability. Another explanation is that attempted compensation present in patients without KS is no longer effective in patients with KS. A previous study conducted in WE patients showed compromised brain synchronisation within the PC (i.e. mammillothalamic connectivity).^23^ Oh *et al*.^24^ observed hypoconnectivity in WE patients in several preselected cerebellar regions. These studies focused on *a priori* regions (seed-based) and investigated rs-FC in WE and not at the chronic stage of KS, as in the present study. Although there are similarities between the patterns of rs-FC abnormalities in WE and KS patients, we cannot conclude regarding the specificities of KS compared with WE.

Beyond its impact on episodic memory abilities, abnormal rs-FC between regions of the PC contributes, in AUD with and without KS, to executive deficits as revealed in the present study, but also to visuospatial impairments,^66^ social cognition^67^ or motor deficiencies.^68^ Consequently, impairment of this circuit in AUD with and without KS leads to changes in daily living skills and a possible loss of autonomy.

This study examined for the first time the effect of AUD on rs-FC in patients with and without KS. Our results point at an attempt towards compensation mechanism through resting-state hyperconnectivity between hippocampi and cerebellar cruses, part of the FCC and PC respectively. However, this process seems to remain insufficient, at the group level, as evidenced by systematic lower cognitive performance in patients with AUD and KS than in HC. Rs-FC dysfunction within hippocampal regions seem to play a role in the massive amnesia of KS^69^ but may also contribute to other cognitive impairments such as executive functions. The attempted compensation mechanism observed in patients with AUD was not present in patients with KS probably due to massive structural alterations observed in this pathology. These results offer new insights into the neurobiological mechanisms underlying AUD with and without KS. Clinically, the identification of biomarkers provides information on the severity of AUD and is critical for identifying AUD patients potentially at risk of KS. However, further structural and functional imaging studies are needed to confirm these findings, ideally in a larger group of patients with KS, even though such a very well documented cohort is extremely rare. Further investigations into the relationship between FDG-PET glucose metabolism, intrinsic functional connectivity and cognitive burden would also be relevant to establish the prevailing pathophysiological mechanisms. Glucose hypermetabolism could reflect a mechanism of maladaptive plasticity as shown in the cerebellum of AUD patients without KS^70^ where hypermetabolism does not compensate cognitive deficits of AUD patients. In KS patients, an FDG-PET study by Maillard *et al*.^71^ also revealed hypermetabolism in the cerebellum and parts of the hippocampus and cingulate cortex compared to HCs during the first year of abstinence. Taken together, these results corroborate the maladaptive cerebello-hippocampal hyperconnectivity found in our study.

## Supplementary Material

fcae294_Supplementary_Data

## Data Availability

All data used within this study will be made available to research groups seeking to reproduce our results. A script for converting imaging data to the BIDS format is available in the Supplementary material.
