# Supplementary material for: Compensation patterns and altered functional connectivity in alcohol use disorder with and without Korsakoff's syndrome
Source: Brain Commun. 2024 Sep 20;6(5):fcae294. doi: 10.1093/braincomms/fcae294 (PMC11414044; doi:10.1093/braincomms/fcae294)
Supplement: fcae294_Supplementary_Data [file fcae294_supplementary_data.docx]

# **Supplementary Material**

**Supplementary Fig. 1: Steps of resting-state functional connectivity data preprocessing and statistical analyses.**


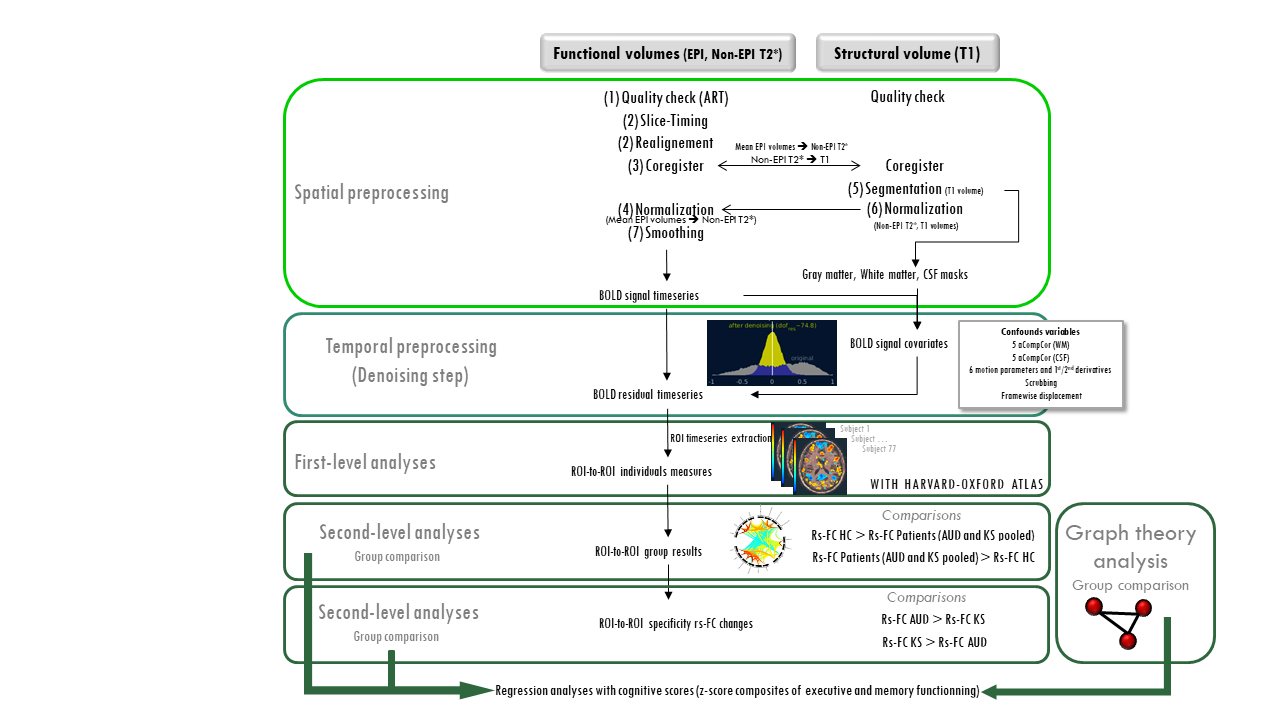


The light green box represents the spatial preprocessing step. The medium green box represents the denoising step. The dark green boxes represent first-and-second-level statistical analyses. AUD: Patients with Alcohol Use Disorder without Korsakoff syndrome, BOLD: Blood Oxygenation Level Dependent, CSF: cerebrospinal fluid, HC: Healthy controls, KS: patients with Korsakoff’s syndrome, ROI: region of interest, Rs-FC: resting-state functional connectivity, WM: white matter.

**Supplementary Fig. 2: Regions of interest for the fronto-cerebellar (blue) and Papez (yellow) circuits.**


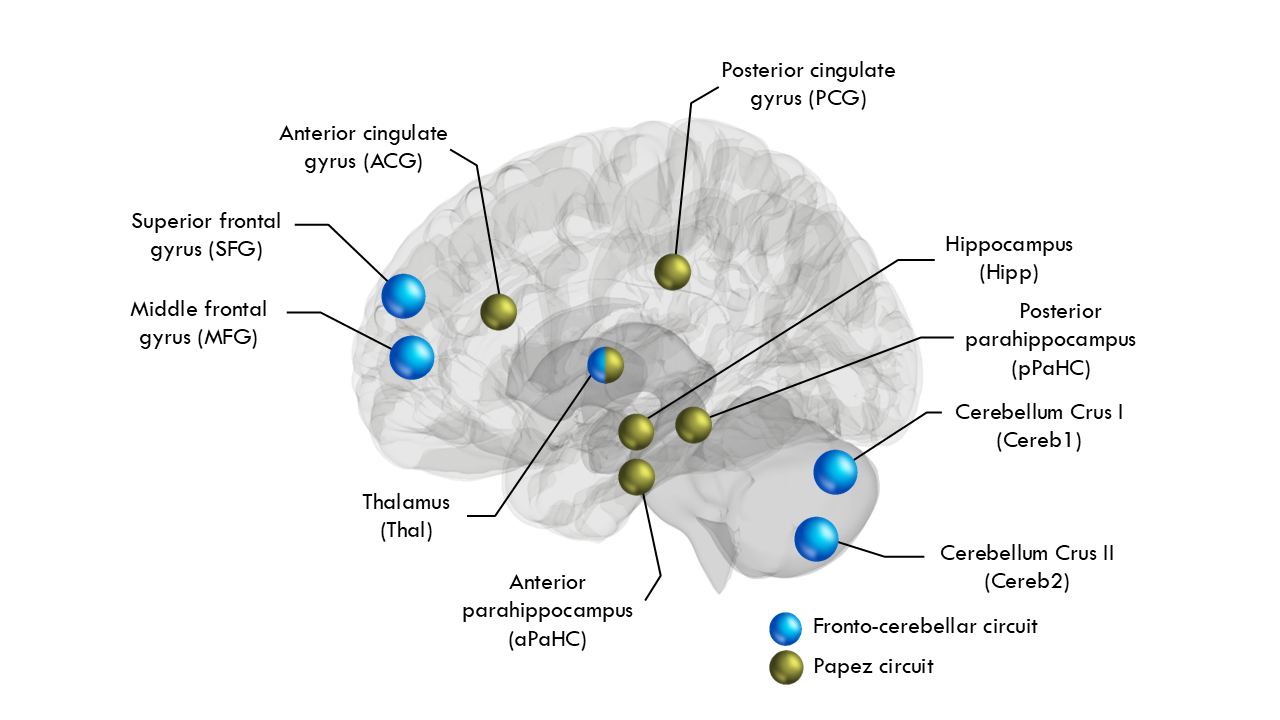


**Supplementary Fig. 3: Significant pairwise resting-state functional connectivity differences between (1) AUD patients compared to the HC group, and (2) KS patients compared to the HC group**

(1)


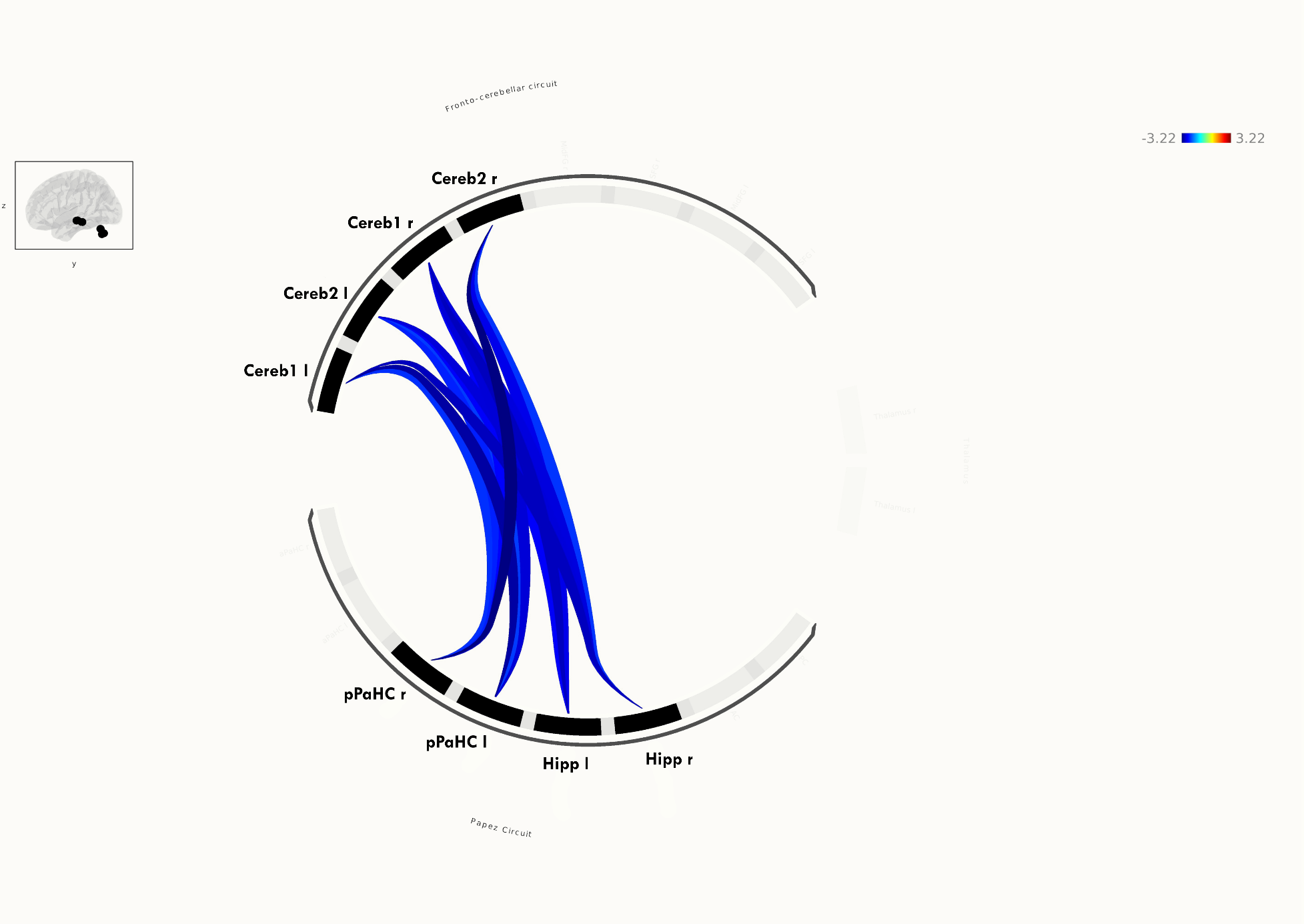


Significant ROI-to-ROI comparisons with a two-sample t-test between AUD patients (n = 32) and healthy controls (n = 31). Blue links represent hyperconnectivity in patients (significant gain of functional connectivity, AUD patients > healthy controls). Results are reported at *p* < .05 FWE.

Abbreviations are: cerebellum Crus I (Cereb1), and cerebellum Crus II (Cereb2) included in the fronto-cerebellar circuit; hippocampus (Hipp), anterior parahippocampal cortex (aPaHC), posterior parahippocampus (pPaHC) included in the Papez circuit.

(2)


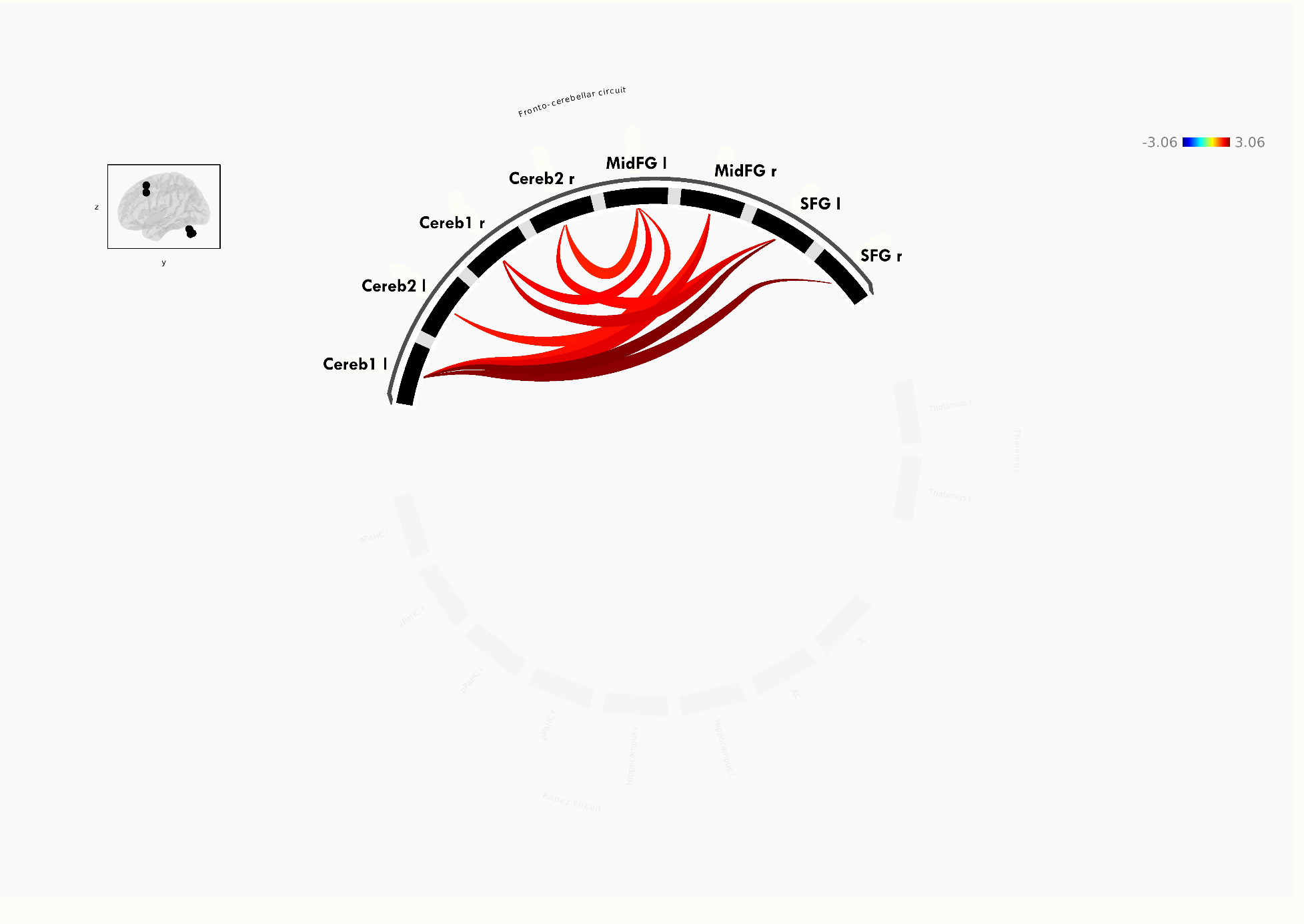


Significant ROI-to-ROI comparisons with a two-sample t-test between KS patients (n = 14) and healthy controls (n = 31). Red links represent hypoconnectivity in patients (significant loss of functional connectivity, Healthy controls > KS patients). Results are reported at *p* < .05 FWE.

Abbreviations are: cerebellum Crus I (Cereb1), cerebellum Crus II (Cereb2), middle frontal gyrus (MidFG), superior frontal gyrus (SFG) included in the fronto-cerebellar circuit.

**Supplementary Table 1**: Table of significant pairwise functional for negative correlations (Patients [AUD and KS pooled] > Healthy controls). Results are obtained with ROI-to-ROI analyses at *p* FWE < 0.05.

| ROI-to-ROI | Statistic *t*(73) | Box plot |
| --- | --- | --- |
| Negative correlations (Patients > Healthy controls) | | |
| pPaHC r – Cereb2 r | -3.14 | 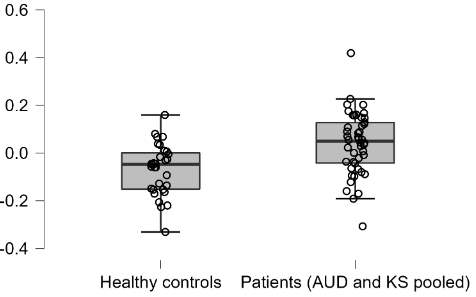 |
| aPaHC r – Cereb2 l | -3.00 | 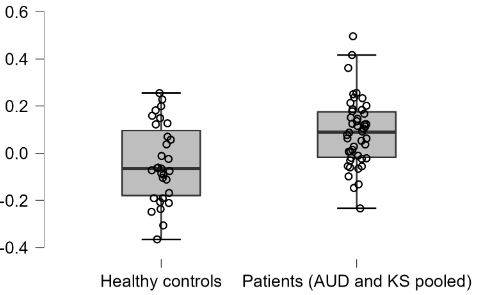 |
| aPaHC r – Cereb2 r | -2.95 | 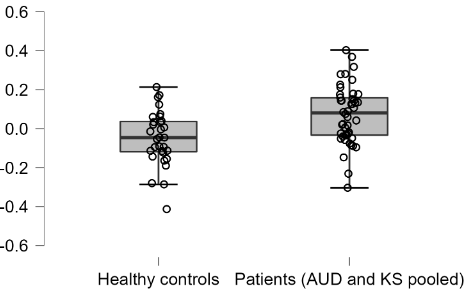 |
| pPaHC l – Cereb1 l | -2.74 | 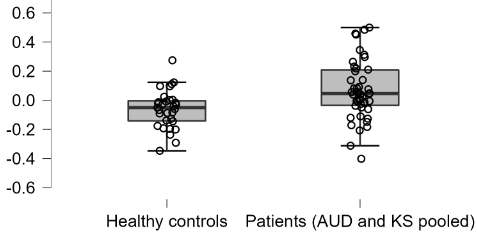 |
| Hipp r – Cereb1 r | -2.60 | 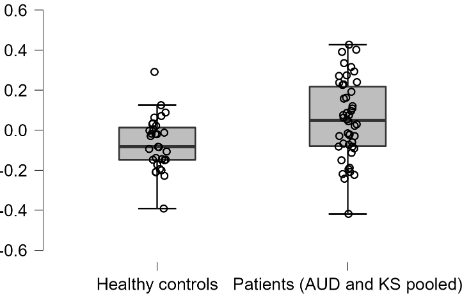 |
| Hipp r – Cereb2 l | -2.56 | 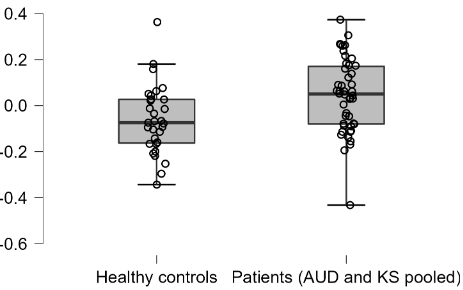 |
| pPaHC l – Cereb1 r | -2.55 | 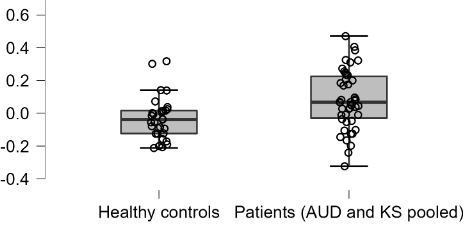 |
| pPaHC r – Cereb1 r | -2.54 | 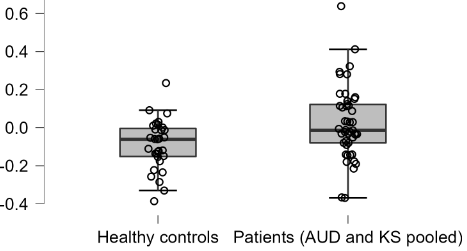 |
| Hipp r – Cereb1 l | -2.47 | 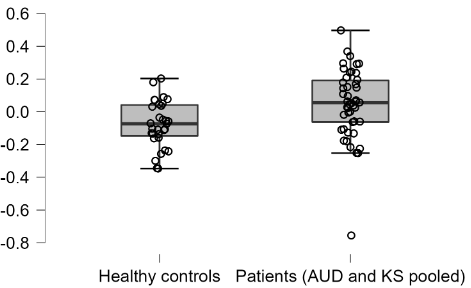 |
| Hipp l – Cereb1 r | -2.46 | 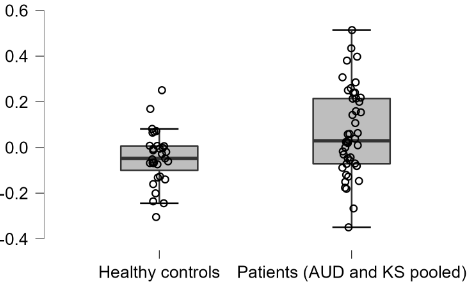 |
| pPaHC r – Cereb2 l | -2.41 | 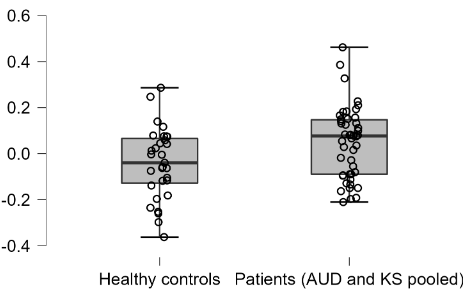 |
| Hipp l – Cereb2 l | -2.39 | 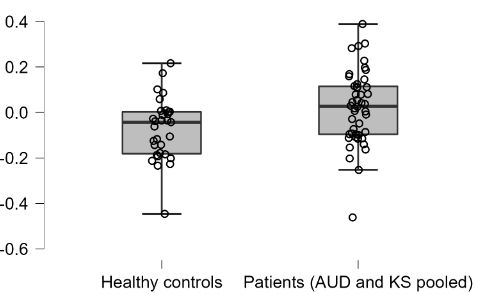 |
| pPaHC l – Cereb2 l | -2.30 | 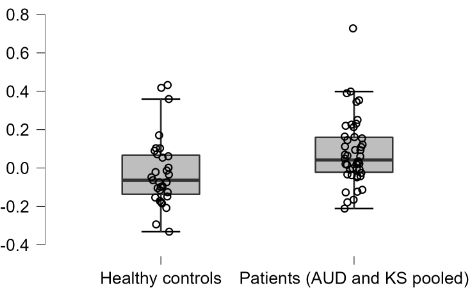 |
| aPaHC r – Cereb1 l | -2.27 | 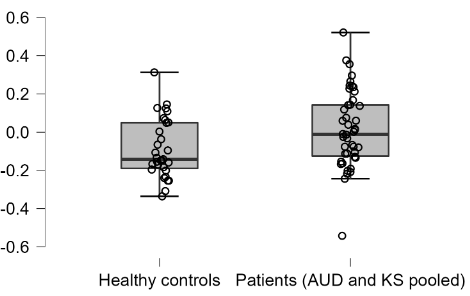 |
| aPaHC r – Cereb1 r | -2.24 | 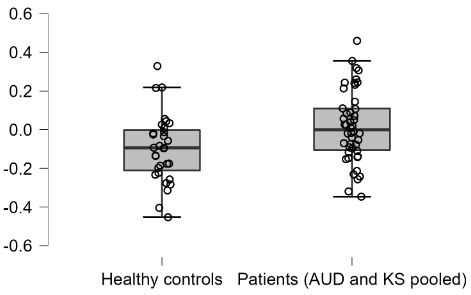 |
| Hipp l – Cereb2 r | -2.17 | 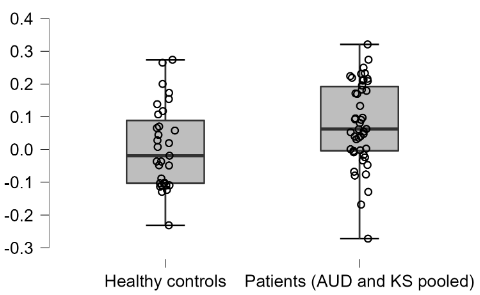 |
| pPaHC r – Cereb1 l | -2.09 | 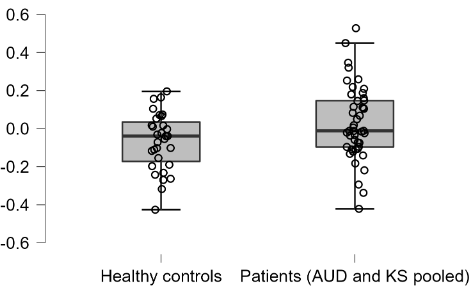 |
| aPaHC l – Cereb2 l | -2.01 | 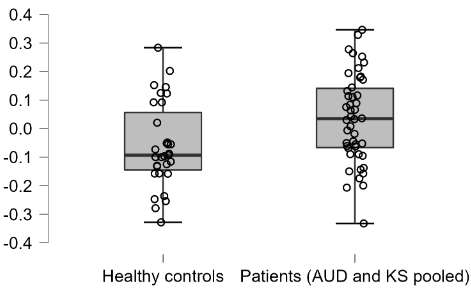 |

Abbreviations: cerebellum Crus I (Cereb1), and cerebellum Crus II (Cereb2) included in the fronto-cerebellar circuit (blue spheres); hippocampus (Hipp), anterior parahippocampus (aPaHC), posterior parahippocampus (pPaHC) are part of the Papez circuit (yellow spheres)

**Supplementary Table 2**: Table of significant pairwise functional for negative correlations (AUD patients > KS patients). Results are obtained with ROI-to-ROI analyses at *p* < 0.001 uncorrected.

| ROI-to-ROI | Statistic *t*(42) | Box plot |
| --- | --- | --- |
| Positive correlations (AUD patients > KS patients) | | |
| Hipp_r – pPaHC_r | 5.35 | 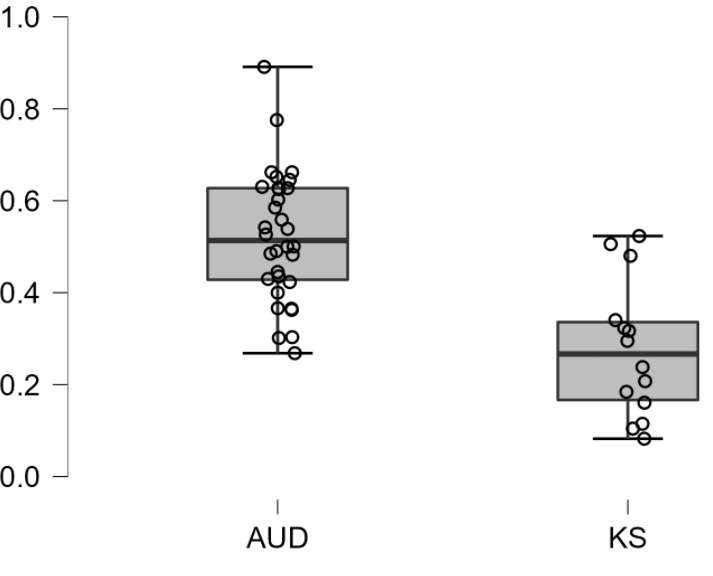 |
| Hipp_l – pPaHC_r | 4.02 | 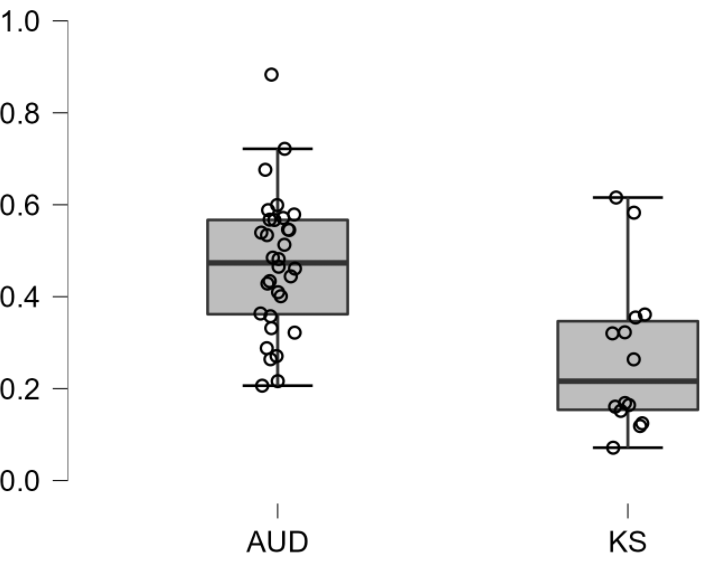 |
| pPaHC_r – aPaHC_r | 3.31 | 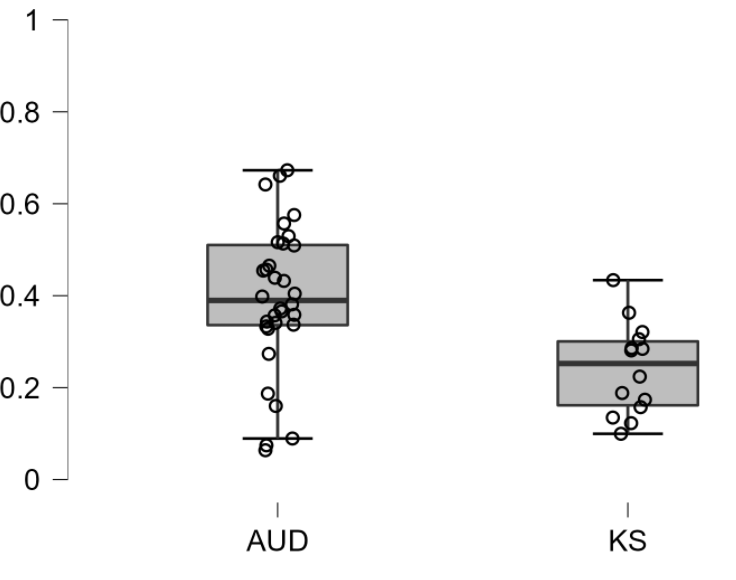 |
| pPaHC_r – aPaHC_l | 2.905 | 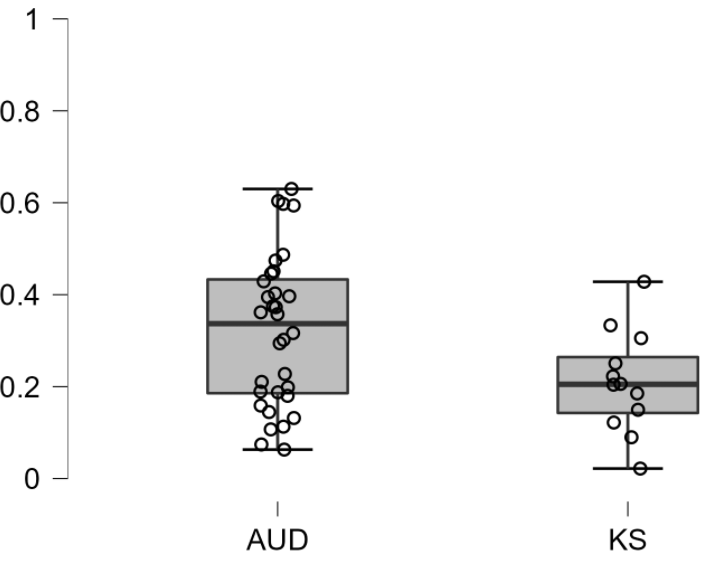 |
| Hipp_r – Hipp_l | 2.759 | 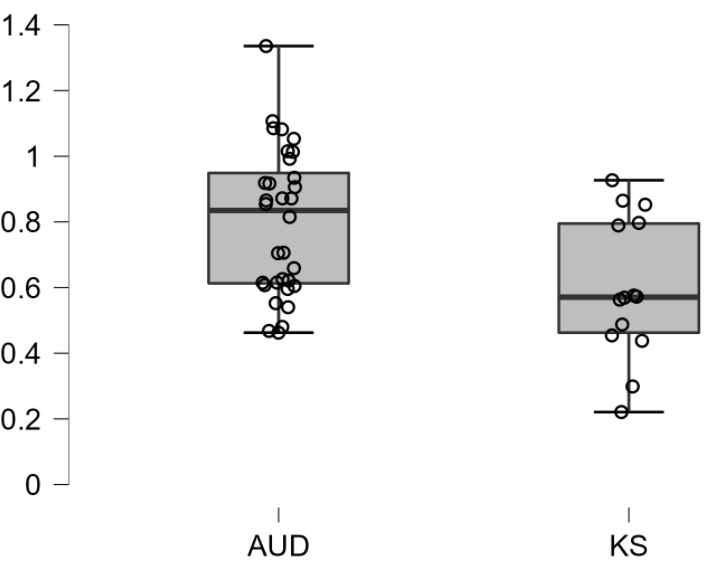 |
| Hipp_r – pPaHC_l | 2.752 | 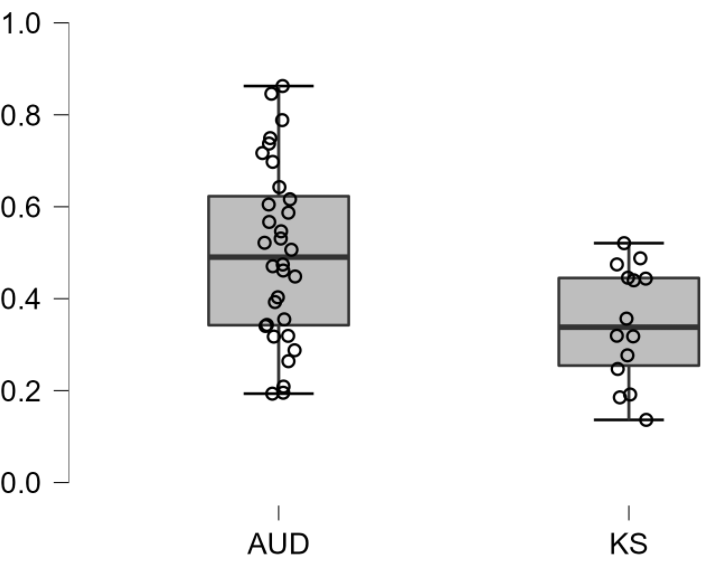 |
| Hipp_r – aPaHC_r | 2.750 | 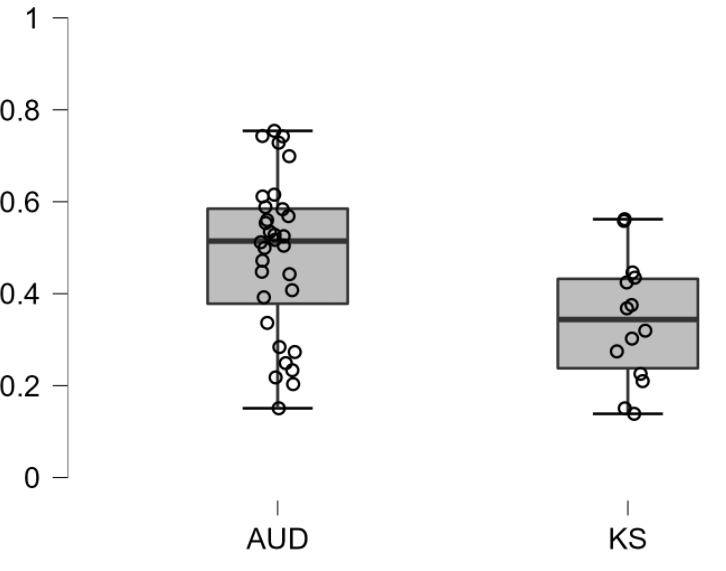 |
| pPaHC_l – pPaHC_r | 2.567 | 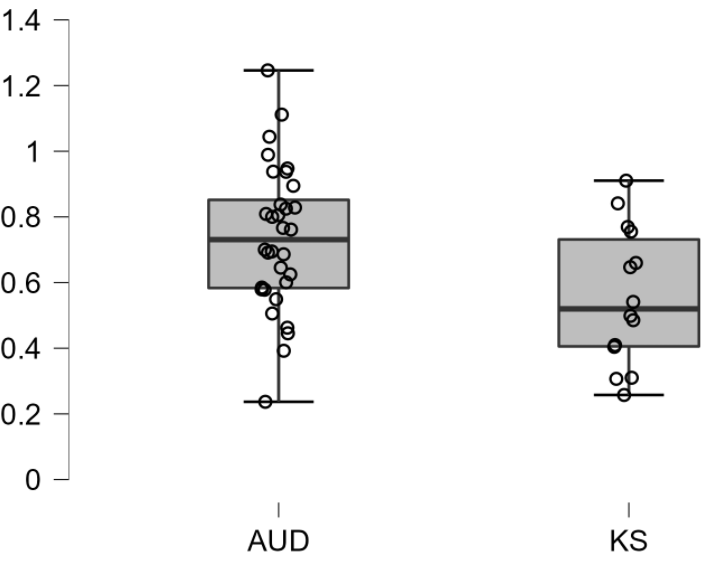 |
| Hipp_r – aPaHC_l | 2.388 | 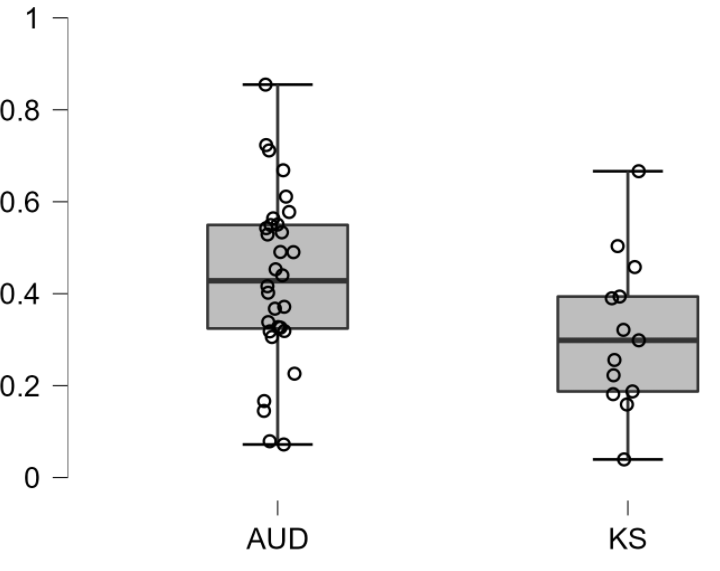 |
| Hipp_l – pPaHC_l | 2.250 | 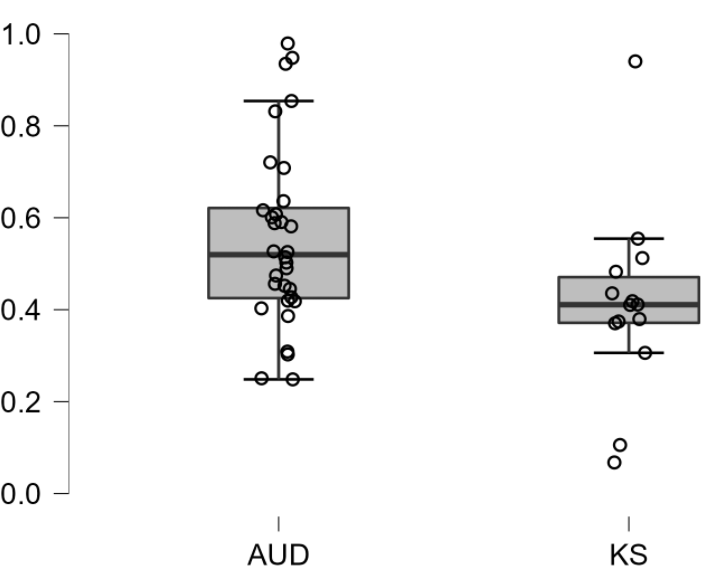 |
| pPaHC_l – aPaHC_l | 2.187 | 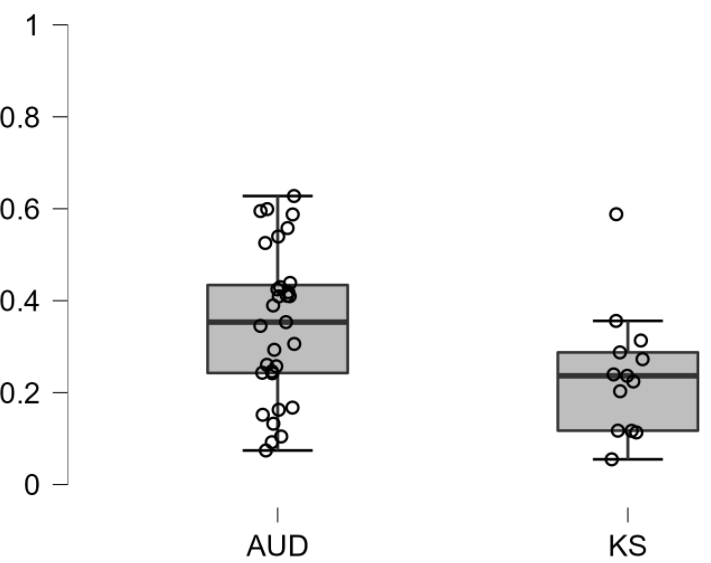 |

Abbreviations: hippocampus (Hipp), anterior parahippocampus (aPaHC), posterior parahippocampus (pPaHC) are part of the Papez circuit (yellow spheres)

**Supplementary Table 3:** Table of the correlations between pairwise resting-state functional hyperconnectivities in AUD and KS patients pooled together and global efficiency of FCC. Results are presented at *p* < 0.05, two-tailed.

| Roi to ROI |  |  |
| --- | --- | --- |
| aPaHC r and Cereb2 l | r | -0.429 |
|  | p-value | 0.003* |
| aPaHC r and Cereb2 r | r | -0.374 |
|  | p-value | 0.010* |
| pPaHC l | r | -0.380 |
|  | p-value | 0.009* |
| Hipp r and Cereb1 r | r | -0.315 |
|  | p-value | 0.033* |
| Hipp r and Cereb2 l | r | -0.257 |
|  | p-value | 0.084 |
| pPaHC l and Cereb1 r | r | -0.290 |
|  | p-value | 0.051 |
| pPaHC r and Cereb1 r | r | -0.340 |
|  | p-value | 0.021* |
| Hipp r and Cereb1 l | r | -0.350 |
|  | p-value | 0.017* |
| Hipp l and Cereb1 r | r | -0.216 |
|  | p-value | 0.149 |
| Hipp l and Cereb2 l | r | -0.310 |
|  | p-value | 0.036* |
| aPaHC r and Cereb1 l | r | -0.344 |
|  | p-value | 0.019* |
| pPaHC r and Cereb1 l | r | -0.388 |
|  | p-value | 0.008* |
| aPaHC l and Cereb2 l | r | -0.443 |
|  | p-value | 0.002* |

Abbreviations: cerebellum Crus I (Cereb1), and cerebellum Crus II (Cereb2) included in the fronto-cerebellar circuit (blue spheres); hippocampus (Hipp), anterior parahippocampus (aPaHC), posterior parahippocampus (pPaHC) are part of the Papez circuit (yellow spheres)

*: significant at p<.05

**Supplementary material available : Script to put files in BIDS format**

#!/bin/bash

#to execute : ~/Files/bids_name.sh

# to modify: base dir, locimg, group, suj

locimg="</path>" #directory source (input)

basedir="</path>" #directory fmriprep (output)

#create folder <name>

cd $basedir

mkdir <folder name>

filename=(patient1 patient2 patient3…)

#subject order corresponds to that of the sub-xx in the folders

for suj in patient1 patient2 patient3…

do

echo

echo

echo

echo

cd $basedir/datasetSK

idx=`echo $suj | tail -c 3`

echo processing subject $suj corresponding to sub-$idx

mkdir sub-$idx

mkdir sub-$idx/anat

mkdir sub-$idx/func

mkdir sub-$idx/dwi

echo repertoire T1 $locimg/$suj/${filename[$idx-1]}/${filename[$idx-1]}"_s_0"?"_anat_t1.nii"

echo repertoire output T1 $basedir/datasetSK/sub-$idx/anat/sub-$idx"_T1w.nii"

echo

cp $locimg/$suj/${filename[$idx-1]}/${filename[$idx-1]}"_s_0"?"_anat_t1.nii" $basedir/datasetSK/sub-$idx/anat/sub-$idx"_T1w.nii" #copy paste and modify T1's name

echo repertoire T2 $locimg/$suj/${filename[$idx-1]}/${filename[$idx-1]}"_s_0"?"_anat_t2_HPC.nii"

echo repertoire output T2 $basedir/datasetSK/sub-$idx/anat/sub-$idx"_T2w.nii"

echo

cp $locimg/$suj/${filename[$idx-1]}/${filename[$idx-1]}"_s_0"?"_anat_t2_HPC.nii" $basedir/datasetSK/sub-$idx/anat/sub-$idx"_T2w.nii" #copy paste and modify T2's name

echo repertoire FLAIR $locimg/$suj/${filename[$idx-1]}/${filename[$idx-1]}"_s_0"?"_FLAIR_VISTA.nii"

echo repertoire output FLAIR $basedir/datasetSK/sub-$idx/anat/sub-$idx"_FLAIR.nii"

echo

cp $locimg/$suj/${filename[$idx-1]}/${filename[$idx-1]}"_s_0"?"_FLAIR_VISTA.nii" $basedir/datasetSK/sub-$idx/anat/sub-$idx"_FLAIR.nii"

echo repertoire rest $locimg/$suj/${filename[$idx-1]}/${filename[$idx-1]}"_s_0"?"_repos.nii"

echo repertoire output rest $basedir/datasetSK/sub-$idx/func/sub-$idx"_task-rest_bold.nii"

echo

cp $locimg/$suj/${filename[$idx-1]}/${filename[$idx-1]}"_s_0"?"_repos.nii" $basedir/datasetSK/sub-$idx/func/sub-$idx"_task-rest_bold.nii" #idem fonc

echo repertoire dwi $locimg/$suj/${filename[$idx-1]}/${filename[$idx-1]}"_s_0"?"_DTI_PMS32op_iso_2mm.nii"

echo repertoire output dwi $basedir/datasetSK/sub-$idx/dwi/sub-$idx"_dwi.nii"

echo

cp $locimg/$suj/${filename[$idx-1]}/${filename[$idx-1]}"_s_0"?"_DTI_PMS32op_iso_2mm.nii" $basedir/datasetSK/sub-$idx/dwi/sub-$idx"_dwi.nii"

done

#sub-idx: idx= index du sujet

idx=9

echo all done
